# Modulation of cellular transcriptome and proteome composition by azidohomoalanine—implications on click chemistry–based secretome analysis

**DOI:** 10.1007/s00109-023-02333-4

**Published:** 2023-05-26

**Authors:** Friederike Kirschner, Danielle Arnold-Schild, Christian Leps, Mateusz Krzysztof Łącki, Matthias Klein, Yannic Chen, Annekathrin Ludt, Federico Marini, Can Kücük, Lara Stein, Ute Distler, Malte Sielaff, Thomas Michna, Kristina Riegel, Krishnaraj Rajalingam, Tobias Bopp, Stefan Tenzer, Hansjörg Schild

**Affiliations:** 1 Institute of Immunology, University Medical Center Mainz, Langenbeckstrasse 1, 55131 Mainz, Germany; 2 Institute of Medical Biostatistics, Epidemiology and Informatics, University Medical Center of the Johannes Gutenberg University Mainz, Mainz, Germany; 3 Helmholtz Institute Translational Oncology, Obere Zahlbacher Straße 63, 55131 Mainz, Germany; 4 Cell Biology Unit, University Medical Center of the Johannes Gutenberg University Mainz, Mainz, Germany; 5 Research Center for Immunotherapy (FZI), Medical Center of the Johannes Gutenberg University Mainz, Mainz, Germany; 6 University Cancer Center Mainz, Medical Center of the Johannes Gutenberg University Mainz, Mainz, Germany; 7 German Cancer Consortium (DKTK), Mainz, Germany

**Keywords:** Tumor micromilieu, Metabolic labeling, Transcriptome, Proteome, Secretome

## Abstract

**Abstract:**

The analysis of the secretome provides important information on proteins defining intercellular communication and the recruitment and behavior of cells in specific tissues. Especially in the context of tumors, secretome data can support decisions for diagnosis and therapy. The mass spectrometry–based analysis of cell-conditioned media is widely used for the unbiased characterization of cancer secretomes in vitro. Metabolic labeling using azide-containing amino acid analogs in combination with click chemistry facilitates this type of analysis in the presence of serum, preventing serum starvation-induced effects. The modified amino acid analogs, however, are less efficiently incorporated into newly synthesized proteins and may perturb protein folding. Combining transcriptome and proteome analysis, we elucidate in detail the effects of metabolic labeling with the methionine analog azidohomoalanine (AHA) on gene and protein expression. Our data reveal that 15–39% of the proteins detected in the secretome displayed changes in transcript and protein expression induced by AHA labeling. Gene Ontology (GO) analyses indicate that metabolic labeling using AHA leads to induction of cellular stress and apoptosis-related pathways and provide first insights on how this affects the composition of the secretome on a global scale.

**Key messages:**

- Azide-containing amino acid analogs affect gene expression profiles.
- Azide-containing amino acid analogs influence cellular proteome.
- Azidohomoalanine labeling induces cellular stress and apoptotic pathways.
- Secretome consists of proteins with dysregulated expression profiles.

**Supplementary Information:**

The online version contains supplementary material available at 10.1007/s00109-023-02333-4.

## Introduction

The development of tumors is a multistep process in which healthy cells of the body develop into uncontrolled growing tumor cells. Tumors, however, are much more than just malignant transformed cells alone. They consist of a heterogeneous collection of infiltrating and resident host cells, as well as extracellular matrix and secreted factors. The sum of all these components constitutes the tumor microenvironment (TME) [1]. Understanding the complex biology of the TME has high potential to reveal attractive strategies to block tumor growth and metastasis by targeting particular components of the TME and achieve durable therapeutic efficacy [2]. The detailed cellular composition of the TME varies between different tumor types but in general contains endothelial cells, fibroblasts, and mesenchymal stromal cells, as well as cells of the adaptive (T and B cells) and innate (macrophages, dendritic cells, neutrophil granulocytes, NK cells) immune system. All these cells constantly secrete proteins and small molecule mediators to orchestrate intercellular communication and other physiological processes [3, 4]. The totality of all secreted substances of a (tumor) cell is termed the secretome and is of high scientific importance as the TME is strongly influenced by secreted substances such as cytokines and chemotactic and growth factors.

The secretome released by tumor cells displays an altered composition compared to the normal tissue from which they are derived [5]. It contains a pathophysiological composition of cytokines, chemokines, hormones, metabolites, and growth factors involved in cell–cell communication, angiogenesis, hypoxia, metastasis, extracellular matrix remodeling, and drug resistance [6], which define a microenvironment that significantly contributes to a mechanism called immune evasion [7]. Therefore, detailed knowledge of the tumor secretome is mandatory for understanding tumor growth and metastasis as well as for the design of specific tumor therapies [8, 9].

Several approaches have been developed over the past years to characterize the secretome of cancer cells. They range on the sample side from the analysis of cancer cell–conditioned media in vitro to biological fluids ex vivo and on the methods side from the use of targeted, e.g., ELISA or proximity extension assay (Olink) analysis to unbiased, e.g., mass spectrometry–based type of protocols [10, 11]. The identification or characterization of tumor-secreted proteins from serum/plasma biomarkers ex vivo is difficult because of the high dynamic range of serum and plasma protein concentrations, and the presence of molecules secreted by different organs, as well as highly abundant proteins such as albumin and immunoglobulins that limit the detection of low abundance proteins [12]. Therefore, analyses of cell-conditioned medium have been widely used to identify tumor-secreted proteins and potential cancer biomarkers [13], despite the fact that tumor cell cultures do not adequately represent tumor tissues [11]. Nevertheless, the significantly reduced complexity facilitates the detection of even low abundance proteins but mostly relies on culture of tumor cells in serum-free medium to reduce the interference with serum proteins [14], thus often inducing serum starvation in the investigated cells. Notably, serum starvation bears the risk of affecting cell viability and function and as a consequence the secretion of proteins [15, 16] or even releasing strictly intracellular components such as proteasomes or ribosomes into the culture medium upon cell death [17]. To overcome these problems, several protocols have been developed employing bioorthogonal metabolic labeling using azide-containing amino acids of cultured cells allowing the capture of newly synthesized proteins through click chemistry from complex mixtures, like serum-containing culture medium [18–20]. This approach is often referred to as the “gold standard” of secretome analysis from cell-conditioned medium, which has been further modified to reduce the number of contaminating proteins observed after the alkyne-based enrichment [21]. However, azide-containing amino acid analogs are incorporated at reduced rates into proteins. This was shown previously in detail for the methionine (Met) analog AHA, which displays an around 400-times reduced translational activity [22].

It is therefore to be expected that the use of AHA affects protein synthesis and secretion. First experiments reveal that the use of AHA resulted in altered expression levels of around 10% of proteins in human primary fibroblasts after 24 h of labeling [23]. To precisely describe and understand the effects of AHA on the behavior of cells, we here performed comparative transcriptome, proteome, and secretome analyses of different tumor cells grown in AHA- or Met-containing media and evaluated the differences in gene and protein expression profiles between normal (Met) and azide-analog containing media. Overall, we observed a significant up- and down-regulation of genes and proteins in response to the replacement of Met by AHA. Depending on the cell line investigated, 15–39% of the proteins detected in the secretome were affected by AHA labeling regarding their expression levels in the transcriptome and proteome. GO analysis revealed that AHA labeling induces the up-regulation of genes and proteins characteristic for cellular stress, regulation of apoptosis, protein translation and folding, and cell proliferation.

In summary, our experiments provide a highly detailed view of AHA effects on cell growth, viability, and AHA-induced perturbations of the cellular transcriptome and proteome. GO analyses point towards AHA-induced cellular stress, induced by protein misfolding and reduced translation rates. These aspects should be kept in mind when interpreting the results of click chemistry–based secretome analyses and suggest that the presence of specific secretome components of interest should be validated by different assays.

## Results

### AHA labeling affects cell growth and viability

**Fig. 1:**
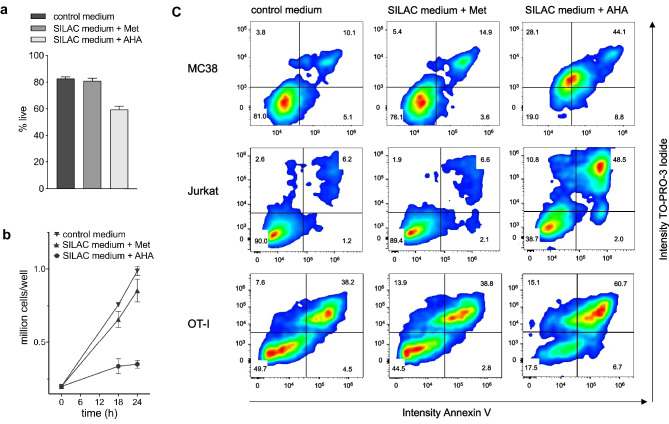
Influence of AHA on cell viability and growth. **a** MC38 cells were grown in control medium and medium containing Met or AHA for 20 h. Cell viability was determined by eFluor780 staining. **b** MC38 cells were cultured in control medium and medium containing Met or AHA. At the indicated time points, live cells were counted using trypan blue staining. **c** Indicated cells were cultured for 20 h in control medium and medium containing Met or AHA. The percentage of living and apoptotic/dead cells was determined by Annexin V and TO-PRO-3 Iodide staining followed by flow cytometry analysis

To analyze in detail the consequences of AHA labeling, we cultured the mouse colon adenocarcinoma cell line MC38 up to 24 h under standard labeling conditions as described in “Methods.” We observed that the replacement of Met by AHA in the culture medium affected cell viability and proliferation (Fig. 1a, b) and induced a shift of the entire cell population into a pre-apoptotic state as indicated by the staining with TO-PRO-3 and Annexin V (Fig. 1c). TO-PRO-3 penetrates cells with compromised membranes characteristic of dead cells and Annexin V detects phosphatidylserine in the plasma membranes of apoptotic cells. This observation was even more pronounced in Jurkat cells and primary T cells from OT-I mice, activated by in vitro stimulation of splenocytes with the cognate synthetic peptide (Fig. 1c). For the Jurkat cells, only 40% of the cells can be found in the live gate after 20 h of AHA labeling and for the OT-I culture, the number of living cells was reduced from around 50% to less than 20%.

**Fig. 2:**
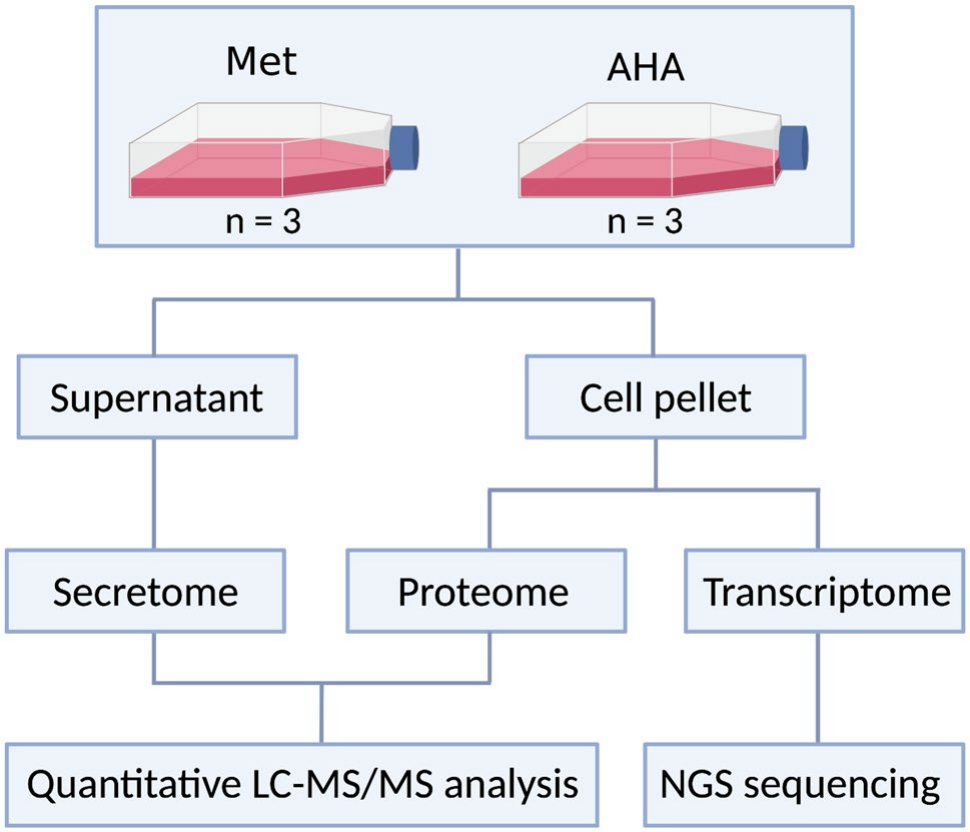
Schematic representation of the experimental workflow used for the transcriptome, proteome, and secretome analysis of the different cell lines. (Created with BioRender.com)

This clear indication of AHA affecting cell viability and growth can be expected to influence the expression of genes, cellular proteostasis, and—as a consequence—also the composition of the proteome. We therefore decided to perform an in-depth analysis of the transcriptome and proteome of different tumor cell lines to further characterize the effects of AHA labeling on protein secretion. Our workflow is depicted in Fig. 2.

### Modification of the transcriptome by AHA labeling

**Fig. 3:**
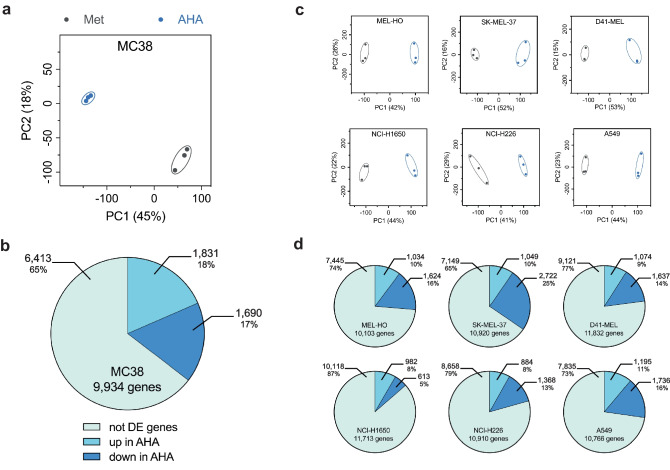
Transcriptome analysis of cell lines cultured in Met or AHA conditions. **a** PCA analysis of MC38 transcriptome after 20 h culture in either Met- or AHA-containing medium. Each dot represents one of three biological replicates. **b** Percentage and number of differentially expressed (DE) genes in MC38 cells after 20 h culture in either Met- or AHA-containing medium. **c** PCA analysis of the transcriptome of different human tumor cell lines after 20 h culture in either Met- or AHA-containing medium. In every plot, each dot represents one of three biological replicates. **d** Percentage and number of DE genes in the different human tumor cell lines after 20 h culture in either Met- or AHA-containing medium

To study the effects of metabolic labeling with AHA on the transcriptome level, we initially investigated the effects on gene expression profiles using MC38 murine colon adenocarcinoma cells. Cells were grown in Met- or AHA-containing medium for 20 h. Proteins and RNA were isolated from the cell pellet as described in “Methods.” RNA sequencing analysis was performed and transcriptome profiles were obtained. Subsequently, we performed a principal component analysis (PCA) of the results from 3 biological replicates of MC38 cells either grown in Met or AHA. Our PCA revealed a very high similarity of the transcriptome within the biological replicates but a noticeable difference between Met and AHA culture conditions (Fig. 3a). Criteria for gene expression and regulation are described in “Methods.” A total of 3521 of the 9934 genes expressed in MC38 cells were differentially regulated in their expression, with 18% being up-regulated and 17% being down-regulated in AHA-containing culture medium after 20 h (Fig. 3b), indicating a profound perturbation of the transcriptome by metabolic labeling with AHA.

To confirm this observation, we extended our analysis using the 3 human melanoma cell lines MEL-HO, SK-MEL-37, and D41-MEL as well as the 3 human lung carcinoma cell lines NCI-H1650, NCI-H226, and A549. Again, PCA of the transcriptome showed remarkable differences between Met and AHA culture conditions (Fig. 3c). Similar to our observation for MC38 cells, 13 to 35% of the genes expressed in the human tumor cells were up- or down-regulated in their expression with a slight tendency for more down-regulated genes (Fig. 3d), confirming the AHA-induced transcriptome perturbation.

### Effects of AHA labeling on proteome composition

**Fig. 4:**
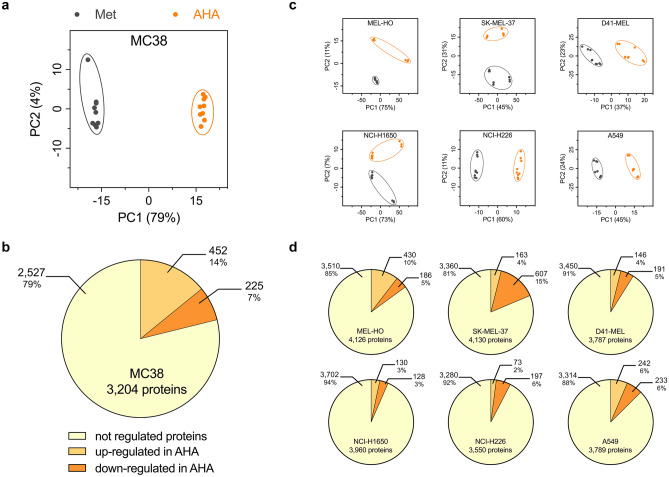
Proteome analysis of cell lines cultured in Met or AHA conditions. **a** PCA analysis of MC38 proteome after 20 h culture in either Met- or AHA-containing medium. The nine dots represent three technical replicates derived from each of the three biological replicates. **b** Percentage and number of differentially regulated proteins in MC38 cells after 20 h culture in either Met- or AHA-containing medium. **c** PCA analysis of the proteome of different human tumor cell lines after 20 h culture in either Met- or AHA-containing medium. The nine dots in every plot represent three technical replicates derived from each of the three biological replicates.** d** Percentage and number of differentially regulated proteins in the different human tumor cell lines after 20 h culture in either Met- or AHA-containing medium

From the identical cell pellet of cultures used above for RNA sequencing analysis, the proteins were isolated and subjected to tryptic digestion and subsequent label-free quantitative mass spectrometry analysis as described in “Methods.” For the analysis of the proteome of MC38 cells, the proteins identified in 3 technical replicates from each of the 3 biological replicates were analyzed with PCA comparing Met and AHA culture conditions. Criteria for protein expression and regulation are described in “Methods.” Technical and biological replicates showed remarkable differences between the two culture conditions (Fig. 4a) and of the total of 3228 proteins detected, 14% were up- and 7% were down-regulated (Fig. 4b). Analyzing the proteins detected in the 3 human melanoma cell lines MEL-HO, SK-MEL-37, and D41-MEL as well as in the 3 human lung carcinoma cell lines NCI-H1650, NCI-H226, and A549, we observed again a clustering of cells grown in Met or AHA conditions after PCA analysis (Fig. 4c). Clustering was more pronounced for NCI-H226, A549, D41-MEL, and SK-MEL-37 cells and less pronounced for NCI-H1650 and MEL-HO cells. The total number of quantified proteins ranged from 3550 to 4152 and up- or down-regulated proteins varied from 6 to 19% (Fig. 4d). These results indicate that metabolic labeling with AHA modifies cellular protein expression profiles, and thus likely also modifies the secretome composition.

### Gene Ontology analysis of genes and proteins with modulated expression after AHA labeling

**Fig. 5:**
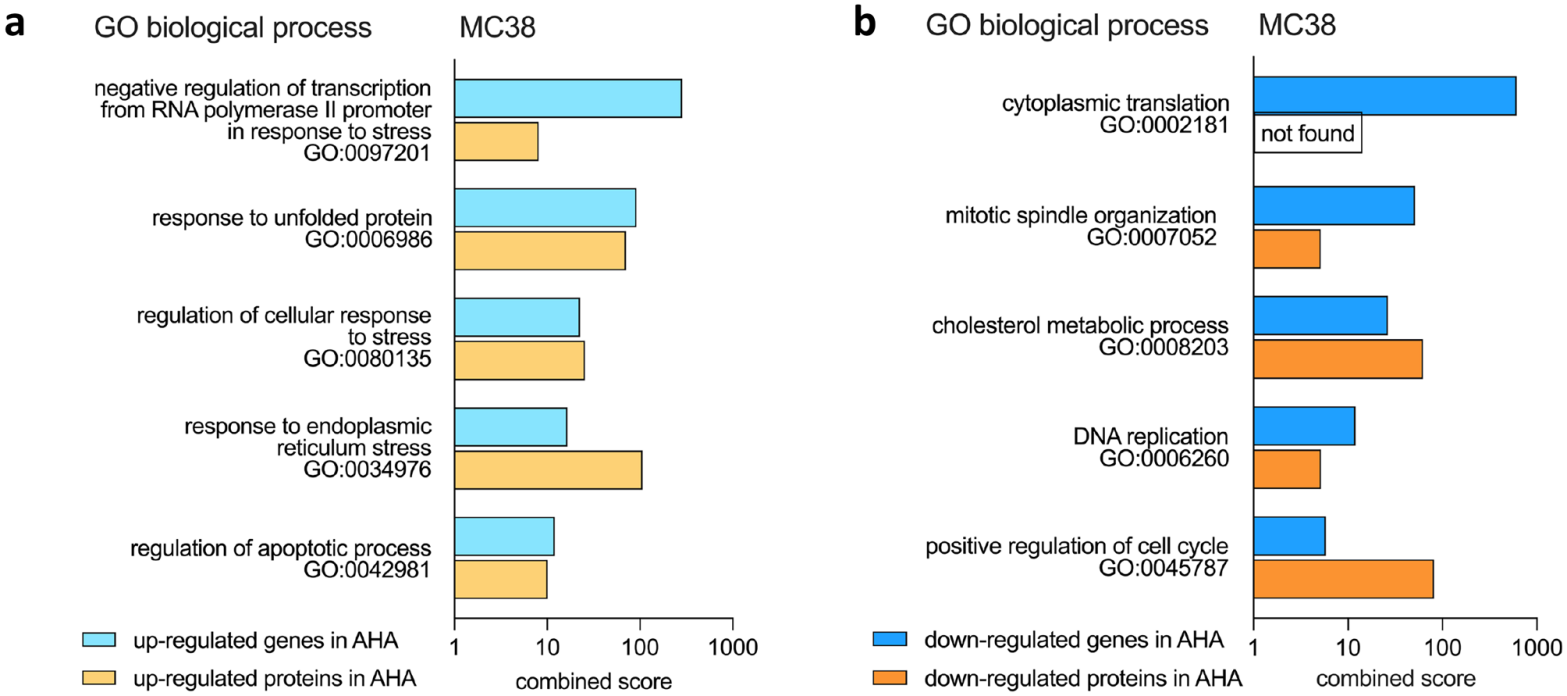
GO analysis of the regulated genes and proteins in MC38 cells cultured in Met or AHA conditions. Transcriptome and proteome profiles of MC38 cells obtained after 20 h culture in either Met- or AHA-containing medium were analyzed using Enrichr software to identify biological pathways affected by AHA labeling. The combined scores for selected GO in the biological processes category are indicated. Pathways identified by up-regulated genes and proteins are shown in **a**, and down-regulated pathways are shown in **b**

**Fig. 6:**
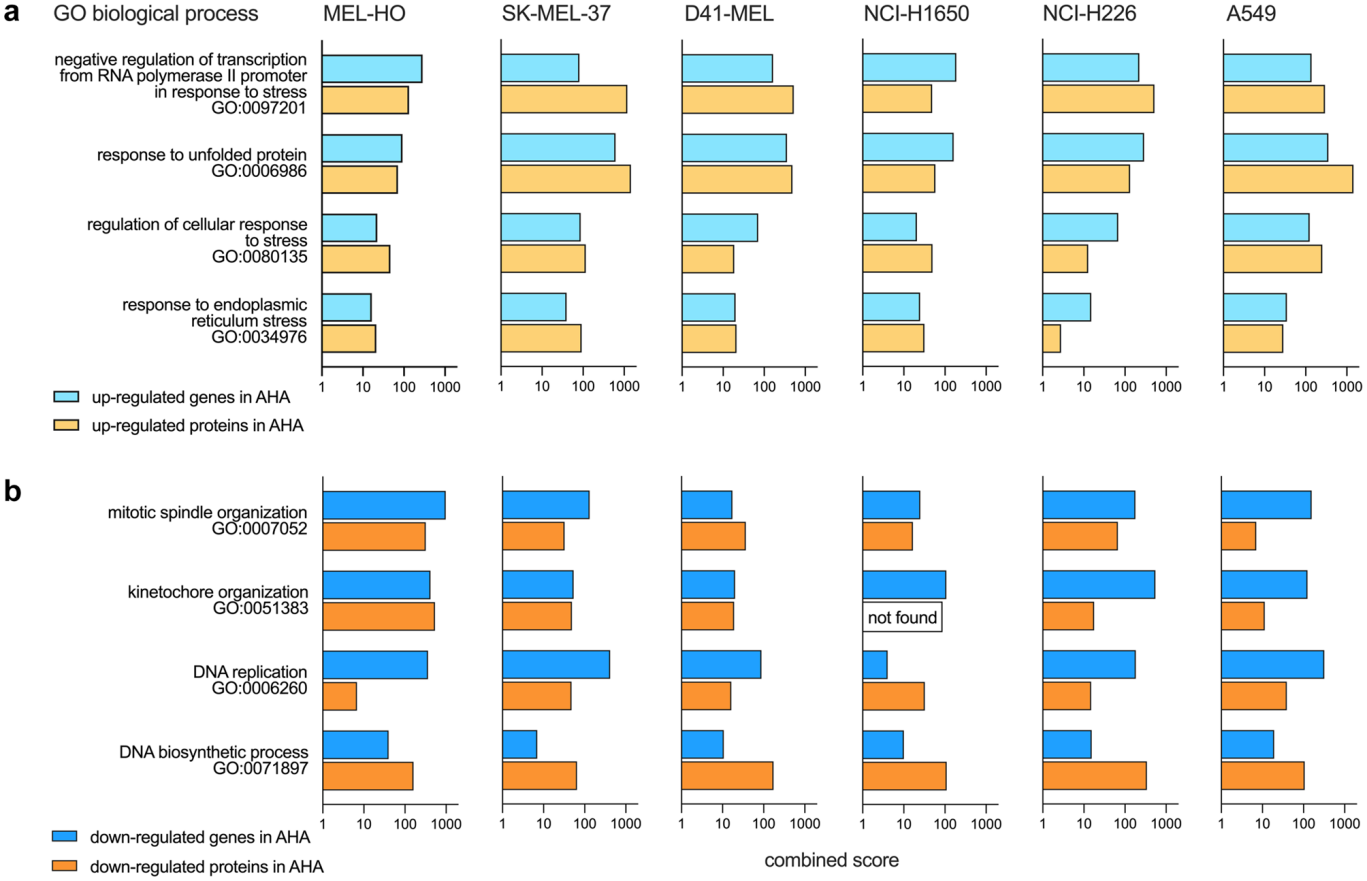
GO analysis of the regulated genes and proteins in the six human tumor cell lines cultured in Met or AHA conditions. Transcriptome and proteome profiles of the different human tumor cell lines obtained after 20 h culture in either Met- or AHA-containing medium were analyzed using Enrichr software to identify biological pathways affected by AHA labeling. The combined scores for selected GO in the biological processes category are indicated. Pathways identified by up-regulated genes and proteins are shown in **a**, and down-regulated pathways are shown in **b**

Having observed that AHA labeling affects the expression levels of a significant number of genes and proteins, we performed GO analyses to identify the affected biological processes and pathways using the Enrichr software tool [24–26]. Initially, analyzing genes and proteins detected in MC38 cells with up- and down-regulated expression after AHA labeling, we observed that pathways correlated with cellular stress and apoptosis were up-regulated (Fig. 5a) whereas pathways involved in cell growth were down-regulated (Fig. 5b), thus indicating that AHA might induce protein misfolding and reduced protein translation. Extending the analysis to the other six cell lines investigated, a highly similar picture emerged, confirming that pathways associated with cellular stress were up-regulated (Fig. 6a) and pathways relevant for cell growth were down-regulated (Fig. 6b).

### Influence of AHA-induced regulation of gene and protein expression on the secretome

**Fig. 7:**
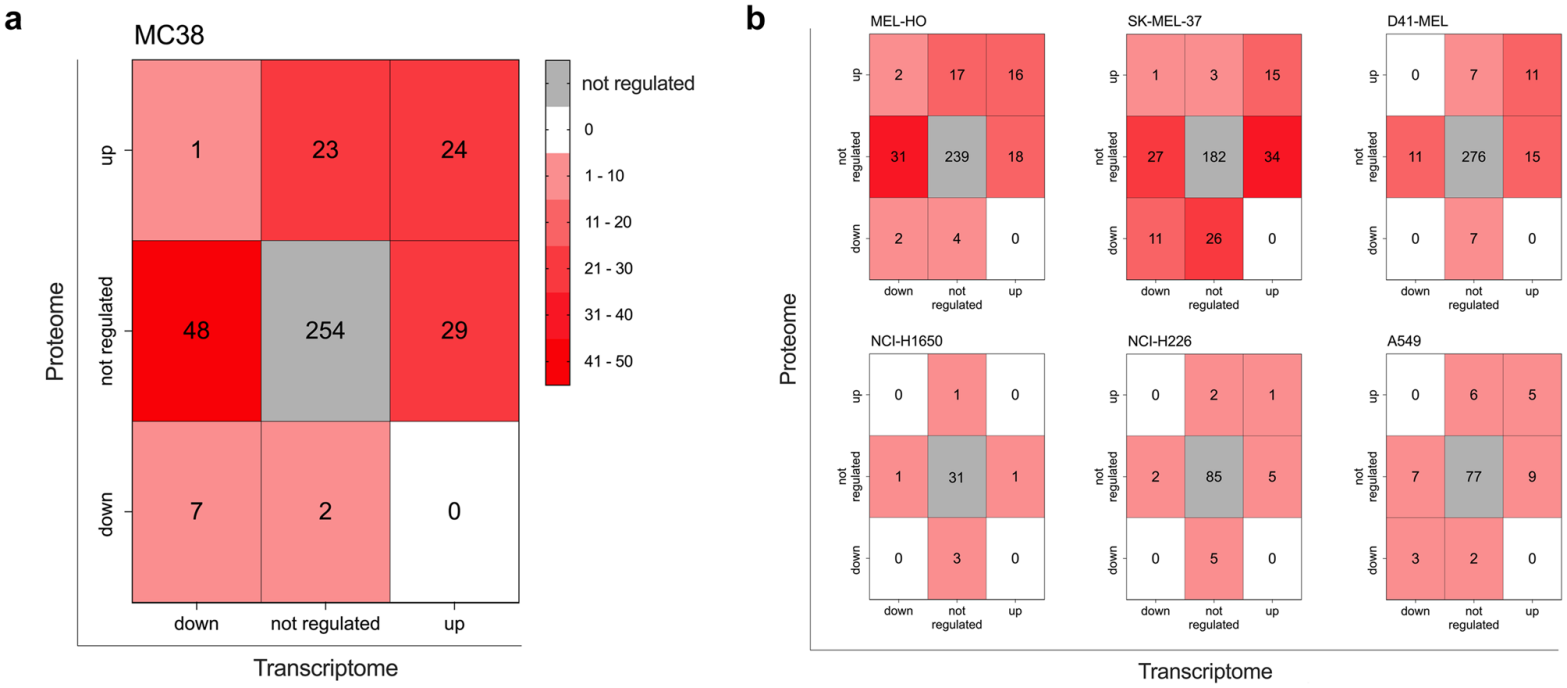
Heatmaps showing the number of secreted proteins with regulated gene and protein expression profile induced by AHA labeling. **a** Distribution of secreted proteins from MC38 cells with regard to their regulation in the transcriptome and proteome expression profile. The colors indicate the number of proteins present in the secretome that show an up-, down-, or not regulated profile in the transcriptome and proteome analysis. The total number of proteins identified in the secretome, which were also detected in transcriptome and proteome analysis, was 388. **b** Analysis as in **a** for the secretome of the six human tumor cell lines. The total number of proteins identified in the secretome and also in transcriptome and proteome analysis was 329 for MEL-HO cells, 299 for SK-MEL-37 cells, 327 for D41-MEL, 37 for NCI-H1650, 100 for NCI-H226, and 109 for A549 cells

To understand the potential consequences of metabolic AHA labeling on the composition of the secretome, the supernatants of MC38 as well as the six human tumor cells grown in Met and AHA conditions were subjected to secretome analyses by mass spectrometry. We identified 521 proteins in the secretome of MC38 cells, based on a protocol similar to Eichelbaum et al. [18] and described in detail in the “Methods” section. From these proteins, 388 were also detected in both transcriptome and proteome analyses (Fig. 7a). Within this group, 254 secreted proteins (corresponding to 66%) showed no up- or down-regulation at the transcriptome and proteome levels. In contrast, 134 secreted proteins (corresponding to 34%) displayed a regulated expression in both transcriptome and proteome analyses.

The same analysis, again with a focus on secreted proteins detected also in the transcriptome and proteome analyses, was performed for the six human tumor cell lines (Fig. 7b). Starting with the different melanoma cell lines, we obtained the following results. The percentage of secreted proteins with regulated expression profiles in the transcriptome and proteome was 27% for MEL-HO cells, 39% for SK-MEL-37 cells, and 15% for D41-MEL cells. Analyzing the secretome of the different lung adenocarcinoma cell lines, we found 16% of the secreted proteins from NCI-H1650 cells, 15% from NCI-H226 cells, and 29% from A549 cells to be regulated in their expression in the transcriptome and proteome analyses. In Supplementary Information SI1, we also provide the information for all proteins identified in the secretome, which were unchanged, up- or down-regulated, including those that could only be detected in the transcriptome or proteome analyses.

## Discussion

The analysis of proteins secreted by cells represents an important tool for the understanding of cellular communication, the behavior of cells in tissues, and—more specifically—the composition of the TME. While workflows to analyze the secretome in serum-containing media have been described, they are hampered by the presence of highly abundant serum proteins, which limit the observable dynamic range and thus the analytical depth of the secretome [27]. To overcome these issues associated with serum-containing media, serum-free culture conditions have been used for secretome analysis and the identification of low abundant secreted proteins [28].

However, cells grown under these conditions often display limited cell growth, show in many cases reduced viability, and will adapt their intracellular pathways to these conditions [20], this potentially skewing the composition of the secretome. To overcome these issues, bioorthogonal metabolic labeling approaches represent an attractive alternative as they allow to grow cells in the presence of serum and to specifically isolate newly synthesized [29–31] and secreted proteins using click chemistry [32] out of complex protein mixtures, e.g., present in the serum-containing culture supernatant. While the analysis of the secretome based on azide-containing amino acid metabolic labeling in combination with click chemistry pioneered by Eichelbaum et al. [18] has distinct advantages, the use of amino acid analogs may induce yet undescribed effects in the investigated cells. Along these lines, it was reported that azide-containing amino acids are incorporated at reduced rates into proteins. This has been illustrated for the Met analog AHA, showing an around 400-times reduced translational activity [22].

These previous observations prompted us to investigate in detail the consequences of AHA labeling on the gene and protein expression profiles and correlate changes with the presence of proteins in the secretome. We observed distinct effects on cell viability (Fig. 1a) and proliferation (Fig. 1b) after 18–24 h of AHA labeling using MC38 cells. Furthermore, AHA labeling for 20 h resulted in the induction of apoptosis ranging from delicate, as observed for MC38 cells, to large effects, as observed for Jurkat cells or primary T cells from OT-I mice (Fig. 1c). This clearly indicates that AHA labeling can affect cell viability with different severities and thereby can be expected to have an impact on the secretome. In line with this, secretome analysis of Jurkat and OT-I T cells after 20 h of AHA labeling revealed, unlike what was observed for MC38 cells (Supplementary Information SI2 a), no significant increase in the number of detected proteins and peptides after click chemistry enrichment in the AHA vs. Met condition (Supplementary Information SI2 b, c). This could indicate that these cells cannot efficiently take up and incorporate AHA and thus experience methionine starvation. For these cell types, alternative secretome workflows using either click chemistry–based enrichment of secreted glycoproteins [33, 34] or click-selective tRNA synthetases [35, 36] might be viable alternatives. However, these approaches either focus on a subset of the secretome or require transfection of the cells of interest.

As MC38 cells were only marginally affected in viability, showed no significant induction of apoptosis (Fig. 1), and allowed for a significant enrichment of AHA-containing proteins (Supplementary Information SI2 a), they appeared to be well suited for AHA-based secretome analysis. We therefore decided to investigate potential AHA effects in detail using the workflow depicted in Fig. 2. Our results indicate that AHA labeling resulted in the up- or down-regulation of more than 30% of the genes identified in transcriptome analysis (Fig. 3a, b) and more than 20% of the proteins identified in proteome analysis (Fig. 4a, b). GO analysis revealed that AHA labeling resulted in the induction of an unfolded protein response and induction of cellular stress as well as inhibition of gene transcription, DNA replication, and the induction of apoptosis (Fig. 5). These findings are well in line with the reduced translational activity of AHA previously described [22] and point towards effects of AHA on protein folding and/or stability. This is supported by our observation that proteins containing higher levels of methionine are enriched in the down-regulated fraction of most cell lines analyzed (Supplementary Information SI3). In addition, only a weak correlation between changes of protein and transcript abundance could be detected (Supplementary Information SI4).

Aligning the regulated expression of genes and proteins with proteins secreted by MC38 cells, we found that more than 30% of the proteins identified in the secretome were up- or down-regulated with regard to their gene and protein expression profile (Fig. 7a). This can be expected to have a significant quantitative and qualitative impact on the composition of the secretome, resulting in an under- or overrepresentation of proteins in the secretome, thus inducing an unwanted and unpredictable bias in the data. We next extended this type of analysis to 3 human lung carcinoma and to 3 human melanoma cell lines. Similar to our observations for MC38 cells, AHA labeling caused an up- or down-regulation of genes ranging from 13 to 35%, resulting in a clear clustering and separation of cells grown in Met or AHA conditions after PCA analysis (Fig. 3c, d). The observed regulation of gene expression affected also the composition of the proteome and again, we found a clustering of cells grown in Met or AHA conditions after PCA analysis and an up- or down-regulation of protein expression varying from 6 to 19% (Fig. 4c, d). Not surprisingly, the level of changes in the gene expression profile correlated with changes in the proteome composition, as nicely exemplified by SK-MEL-37, showing the highest, and NCI-H1650 cells, showing the lowest level of alterations (Figs. 3d and 4d). As observed for MC38 cells, a substantial number of proteins identified in the secretome of the six human tumor cell lines displayed an AHA-induced up- or down-regulated expression profile, ranging from 15 to 39% (Fig. 7b). This strongly suggests that the detected secretome composition is modulated by the AHA labeling. In line with this, the GO analysis of the regulated genes and proteins (Figs. 5 and 6) indicated that AHA might induce protein misfolding and reduced protein translation.

Across all cell lines, over 95% of the proteins that we consider secreted are also present in the Human Cancer Secretome Database [37]. The low number of proteins detected in the secretome of the lung carcinoma cell lines compared to what was identified in the melanoma cell lines might be a consequence of the observed slow growth rate of these cells. As a consequence, these cells might display a lower rate of protein translation and therefore lower numbers of proteins carrying an AHA label, allowing their purification by click chemistry.

To alleviate the effects on the qualitative and quantitative protein composition of the secretome induced by AHA labeling, shorter incubation times may be used, but those will also reduce the amounts of labeled proteins and thus limit the sensitivity of this approach, as it will reduce the amounts of proteins available for click chemistry–based enrichment from serum-containing culture medium. Alternatively, novel approaches employing nanoparticles to compress the dynamic range of biological fluids [38, 39] may also be adapted to analyze the secretome of cells cultured in the presence of serum.

Concludingly, our data describe a profound effect of metabolic labeling using AHA on both transcriptome and proteome level. In addition, not all cell types seem to be able to either take up or incorporate AHA. This became evident during the analysis of proteins secreted by the Jurkat T cell line or primary T cells derived from TCR-transgenic OT-I mice. These cells displayed high rates of cell death (Fig. 1c) and in contrast to, e.g., MC38 cells, showed no significant enrichment of click chemistry–captured proteins (Supplementary Information SI2).

Our results indicate that bioorthogonal metabolic labeling–based analyses targeting newly synthesized [40] and/or secreted proteins should be preceded by detailed analyses regarding cellular viability, the induction of apoptosis, and, if possible, alterations in the gene expression profiles. This will provide helpful insights for adapting existing protocols, e.g., by reducing labeling times or selecting alternative methods, better suited for the cells to be analyzed.

## Methods

### Cell culture

MC38, SK-MEL-37, and A549 cell lines were maintained in DMEM containing 10% FCS, 2 mM glutamine, 1 mM sodium-pyruvate, 100 units/mL penicillin, and 100 μg/mL streptomycin (control medium). The cell lines Jurkat, MEL-HO, D41-MEL, NCI-H226, and NCI-H1650 were cultured in RPMI complemented with 10% FCS, 2 mM glutamine, 1 mM sodium-pyruvate, 100 units/mL penicillin, and 100 μg/mL streptomycin (control medium). MC38 cells were kindly provided by H.-C. Probst (Institute for Immunology, University Medical Center, Johannes Gutenberg University Mainz), NCI-H226 (CRL-5826) and NCI-H1650 (CRL-5883) cells were obtained from ATCC, A549 (ACC 107) and MEL-HO (ACC 62) cells were obtained from the DSMZ-German Collection of Microorganisms and Cell Culture GmbH, D41-MEL cells were kindly provided by Catherine Wölfel (Department of Hematology and Oncology, University Medical Center, Johannes Gutenberg University Mainz), Jurkat cells were kindly provided by Ari Waisman (Institute for Molecular Medicine, University Medical Center, Johannes Gutenberg University Mainz), and SK-MEL-37 cells were obtained from Sigma-Aldrich (SCC262).

OT-I transgenic mice, purchased by Charles River Laboratories, were used to generate primary T cells. OT-I splenocytes were cultured in control medium: RPMI 1640 supplemented with 10% FCS, 2 mM glutamine, 100 units/mL penicillin, 100 μg/mL streptomycin, 50 μM β-mercaptoethanol, in the presence of IL-2 (supernatant of XL-63 cells used at a dilution of 1:250, kindly provided by H.-C. Probst (Institute for Immunology, University Medical Center, Johannes Gutenberg University Mainz)), and peptide (1 μg/mL ovalbumin 257–264) for 5 days. The culture in Met or AHA conditions was performed using the appropriate Stable Isotope Labeling by Amino acids in Cell culture (SILAC) medium without l-leucine, l-arginine, l-lysine, and l-methionine (SILAC DMEM or SILAC RPMI, both purchased by Athena Enzyme Systems) containing all the supplements used in the control medium and supplemented either with 0.8 mM l-leucine, 0.8 mM l-lysine, 0.4 mM l-arginine, and 0.2 mM l-methionine (SILAC medium + Met) or with 0.8 mM l-leucine, 0.8 mM l-lysine, 0.4 mM l-arginine, and 0.1 mM l-AHA (SILAC medium + AHA). All cells were maintained in a humidified incubator with 5% CO_2_ at 37 °C.

### Cell growth and viability

Cell viability was performed using MC38 cells incubated either in control medium or in SILAC medium supplemented with Met or AHA. After 20 h of incubation, cells were fixed and stained using the fixable viability dye eFluor780 (eBioscience) before analyzing live and dead cells using the BD FACSCanto system and the DIVA software. For cell growth determination, 0.2 × 10^6^ MC38 cells were seeded in 6-well plates and grown in control medium or in SILAC labeling medium supplemented with Met or AHA. After 18 and 24 h, cells were harvested and counted microscopically using trypan blue staining for dead cell exclusion. For each condition, three biological replicates were generated. For the Annexin V staining, 1 × 10^6^ MC38, Jurkat, or primary OT-I cells incubated for 20 h in three different media (control medium, SILAC medium + Met, or SILAC medium + AHA) were harvested, washed in Annexin-binding buffer (10 mM Hepes, 140 mM NaCl, and 2.5 mM CaCl_2_), and stained with Annexin V–FITC (BD Biosciences) according to the manufacturer’s protocol for 15 min at 20 °C in the dark. Before analysis, TO-PRO-3 Iodide (Invitrogen) was added to a final concentration of 40 nM and samples were acquired using the Amnis ImageStream MK II and analyzed using the FlowJo software.

### AHA labeling and enrichment of newly synthesized proteins

Secretome analysis was performed using the click chemistry–based approach similar to the method developed by Eichelbaum et al. [18]. Cells were grown in their appropriate control medium to 70% confluency in two T75 flasks (Greiner Bio-One CELLSTAR) for each biological replicate. After removing the medium, cells were washed twice with warm PBS and 7 mL of starvation medium (appropriate SILAC medium with the required supplements as described for the control media of the different cells used, including 0.8 mM l-leucine, but without Met or AHA, l-lysine, and l-arginine) was added. After 30 min of starvation, the medium was removed and cells were cultured either in AHA labeling medium (SILAC medium supplemented with 0.8 mM l-leucine, 0.8 mM l-lysine, 0.4 mM l-arginine, and 0.1 mM l-AHA) or in Met medium (same SILAC medium supplemented with 0.2 mM l-methionine instead of l-AHA). After 20 h labeling, the supernatants were collected, centrifuged for 5 min at 1000 g and 4 °C to remove remaining cells, and concentrated using Amicon Ultra-15 tubes (molecular mass cutoff 3000 Da, Millipore) to 0.25 mL. For all the cell lines and for the primary OT-I cells, three biological replicates were generated. Newly synthesized, AHA-containing proteins were isolated from the concentrated supernatants by performing click chemistry–based enrichment using the Jena Bioscience Click Chemistry Capture Kit according to the manufacturer’s protocol. For both conditions (AHA and Met), supernatants were washed thoroughly to remove unspecifically bound proteins. For subsequent proteomic analysis by mass spectrometry, proteins bound to the beads were reduced, alkylated, and digested with trypsin. For digestion, beads were suspended in 50 µL digestion buffer (50 mM Tris, pH 8, 2 mM CaCl_2_, and 0.1% RapiGest), and 0.5 μg trypsin was added and incubated overnight at 37 °C. The peptide solution was collected and the resin was washed with 50 μL 50 mM ammonium bicarbonate (NH_4_HCO_3_). Both solutions were combined and kept frozen until sample preparation for mass spectrometry analysis.

### RNAseq—sample preparation and biostatistics

After collecting supernatants for secretome analysis, pellets from the same cells grown in both conditions (Met and AHA) were washed twice with PBS and subjected to transcriptome analysis. RNA was purified with the RNeasy Plus Micro Kit according to the manufacturer’s protocol (Qiagen). RNA was quantified with a Qubit 2.0 fluorometer (Invitrogen) and the quality was assessed on a Bioanalyzer 2100 (Agilent) using an RNA 6000 Pico chip (Agilent). Samples with an RNA integrity number (RIN) of ≥ 8 were used for library preparation. Barcoded mRNA-seq cDNA libraries were prepared from 150 ng of total RNA using NEBNext^®^ Poly(A) mRNA Magnetic Isolation Module and NEBNext^®^ Ultra™ II RNA Library Prep Kit for Illumina^®^ according to the manual with a final amplification of 12 PCR cycles. Quantity was assessed using Invitrogen’s Qubit HS assay kit and library size was determined using Agilent’s 2100 Bioanalyzer HS DNA assay. Sequencing was performed on Illumina’s NovaSeq 6000 at Novogene (Cambridge, UK). Raw sequencing reads (approx. 30 mio 150 PE reads per sample) were preprocessed according to the Illumina standard protocol. Sequence reads were trimmed for adapter sequences and further processed using Qiagen’s software CLC Genomics Workbenchv20.0 with CLC’s default settings for RNAseq analysis. Reads were aligned toGRCm38 (file version GRCm38.p6) or GRCh38 (file version nGRCh38.104) genome dependent on cell line with the following settings: mismatch cost = 2; insertion cost = 3; deletion cost = 3; length fraction = 0.8; similarity fraction = 0.8. Detailed tables with expression values TPM, RPKM, total, and unique gene reads for each sample are deposited under the GEO accession number GSE211231. PCA plots were generated using CLC’s tool “PCA for RNA-Seq” with the following filtering and normalization settings: (a) “log CPM” (counts per million) values are calculated for each gene. The CPM calculation uses the effective library sizes as calculated by the TMM normalization. After this, a *Z*-score normalization is performed across samples for each gene: the counts for each gene are mean centered, and scaled to unit variance. Genes or transcripts with zero expression across all samples or invalid values (NaN or + / − infinity) are removed.

For differential expression analysis, in order to filter out non- or low-expressed genes, genes with TPM mean expression ≥ 4 in any group (Met or AHA treated) were used for subsequent analysis. For statistical analysis, CLC’s count based “Empirical analysis of Differential Gene Expression” implementing the “Exact Test” for two-group comparisons developed by Robinson and Smyth [41] was applied for each Met- versus AHA-treated cell line. For the generation of pie charts showing up- and down-regulated genes upon AHA treatment, the gene list of Met vs AHA comparism of each cell line was filtered on absolute fold change ≥ 2 and *p* values less than 0.05, which were considered statistically significant in this study.

### Mass spectrometry—sample preparation

Secretome samples were digested “on-bead” during secretome protocol and subsequently desalted, using a Sep-Pak tC18 µElution Plate (Waters Corporation, Milford, MA) and a vacuum manifold. Purified peptides were lyophilized and reconstituted in 20 µL 0.1% (v/v) formic acid (FA) for LC–MS analysis. Proteome samples were digested according to filter-aided sample preparation (FASP) as described previously [42]. In brief, cell pellets were solved in 500 µL lysis buffer, containing 8 M urea, and disrupted in 15 cycles at 30 s sonification, followed by 30 s break in a Bioruptor (Diagenode, Liège, Belgium). Protein concentration was determined via Pierce 660 nm protein assay (Thermo Fisher Scientific), according to the manufacturer’s protocol and 20 µg protein was reduced using dithiothreitol (DTT), followed by alkylation with iodoacetamide (IAA). DTT was added again, to quench excess IAA. Buffer was exchanged by washing the membrane three times with 50 mM NH_4_HCO_3_ prior to digestion overnight at 37 °C using trypsin at an enzyme-to-protein ratio of 1:50 (w/w). After digestion, peptides were eluted via centrifugation, followed by washing the membrane once again with 50 mM NH_4_HCO_3_. Next, the samples were lyophilized and finally the purified peptides were reconstituted in 20 µL 0.1% (v/v) FA for LC–MS analysis.

### Mass spectrometry—data acquisition

Nanoscale liquid chromatography (nanoLC) of tryptic peptides was performed on an Ultimate 3000 RSLCnano LC system (Thermo Fisher Scientific) equipped with a PEPMAP100 C18 5 µm 0.3 × 5 mm trap (Thermo Fisher Scientific) and an HSS-T3 C18 1.8 μm 75 μm × 250 mm analytical reversed-phase column (Waters Corporation). Mobile phase A was 0.1% (v/v) FA and 3% (v/v) dimethyl sulfoxide (DMSO) in water. Mobile phase B was 0.1% (v/v) FA and 3% (v/v) DMSO in acetonitrile. Peptides were separated running a gradient from 2 to 35% at a flow rate of 300 nL/min at 55 °C over 40 min. Together with wash- and column re-equilibration steps, the total analysis time was 60 min. Eluting peptides underwent mass spectrometric analysis on an Orbitrap Exploris 480 (Thermo Fisher Scientific) in a data-dependent acquisition (DDA) mode targeting the 10 most abundant peptides for fragmentation (Top10). Spray voltage was at 1.8 kV, the funnel RF level at 40, and heated capillary temperature at 275 °C. Full MS resolution was set to 120,000 at *m/z* 200 and full MS automated gain control (AGC) target to 300% with a maximum injection time of 50 ms. Mass range was set to *m/z* 350–1500. The limit of isolated peptide precursors for MS2 scans was set to an ion target of 1 × 10^5^ (AGC target value of 100%) with maximum injection times of 25 ms. Fragment ion spectra were acquired at a resolution of 15,000 at *m/z* 200. Intensity threshold was kept at 1E4. Isolation window width of the quadrupole was set to *m/z* 1.6 and normalized collision energy was fixed at 30%. All data were acquired in profile mode using positive polarity.

### Mass spectrometry—data processing

Acquired raw data were processed in MaxQuant Version 2.0.3.0 [43] with database search performed in the integrated search engine Andromeda. For human cell lines, the UniProt human proteome database (UniProtKB release 2020–3-2_2-0–11, 20,365 entries), including 172 common contaminants, was used and for MC38 and OT-I cell line the UniProt mouse proteome database (UniProtKB release 2020–3-2_2-0–11, 17,033 entries), including 172 common contaminants. Trypsin was specified as enzyme for digestion and a maximum of two missed cleavages per peptide was allowed. Fixed modification was set for carbamidomethyl cysteine and variable modification was set for oxidized methionine. False discovery rate assessment for peptide and protein identification was done using the target-decoy strategy by searching a reverse database and was set to 0.01 for database search in MaxQuant. TOP3 quantification [44] was used to infer protein level quantities.

### Mass spectrometry—statistical analysis

Obtained LFQ intensities were statistically analyzed in Microsoft Excel (2019) by performing two-tailed, unpaired *t*-tests across all technical and biological replicates and subsequent Benjamini–Hochberg correction [45]. The log2 ratio was calculated for each cell line by subtracting the log2 of the average LFQ intensities across all technical and biological replicates in the AHA group from the log2 of the average LFQ intensities across all technical and biological replicates in the methionine group. For entries with no detection in either group (Met or AHA), values were imputed by dividing the minimum intensity available in the corresponding dataset by 2. To define the secretome dataset, we applied a filter to retain only proteins with a log2 ratio ≤  − 1 between Met and AHA conditions after click chemistry–based enrichment. In the proteome data, proteins with a log2 ratio ≤  − 1 or ≥ 1 were considered to be differentially expressed, in AHA or Met condition, respectively. To align secretome, proteome, and transcriptome data, the entry lists of each dataset were used to detect uniquely or commonly present entries (proteins or genes), using R version 4.0.4 (2021–02-15).

### Bioinformatics

Differentially expressed genes between both Met and AHA conditions were subjected to a GO analysis using the Enrichr web application (https://maayanlab.cloud/Enrichr/) [24–26]. The enrichment results in the category biological process were visualized in tables ranking the enriched gene set library terms according to their combined score. Before regulated proteins between Met and AHA treatments were analyzed using the Enrichr software, the UniProt proteins IDs were converted to gene IDs using the SYNGO tool (https://www.syngoportal.org/convert) [46]. GO enrichment results of regulated proteins in the category biological process were listed in tables, allowing visualization of GO terms with the highest combined scores.

## Supplementary Information

Below is the link to the electronic supplementary material.

Supplementary file1 (PDF 668 KB)

Supplementary file2 (PDF 241 KB)

Supplementary file3 (PDF 152 KB)

Supplementary file4 (PDF 280 KB)

Supplementary file5 (XLSX 190 KB)

## Data Availability

Transcriptome data were deposited in Gene Expression Omnibus (GEO) (https://www.ncbi.nlm.nih.gov/geo/) under the accession number GSE211231. Proteome and secretome data were submitted to the jPOST repository protein data bank (https://repository.jpostdb.org) [47] and are available under the accession number JPST001899. In addition, we provide a list of all differentially expressed proteins in Supplementary Information SI5.
