# Supplementary figures and images for: Modulation of cellular transcriptome and proteome composition by azidohomoalanine—implications on click chemistry–based secretome analysis
Source: J Mol Med (Berl). 2023 May 26;101(7):855–67. doi: 10.1007/s00109-023-02333-4 (PMC10300158; doi:10.1007/s00109-023-02333-4)

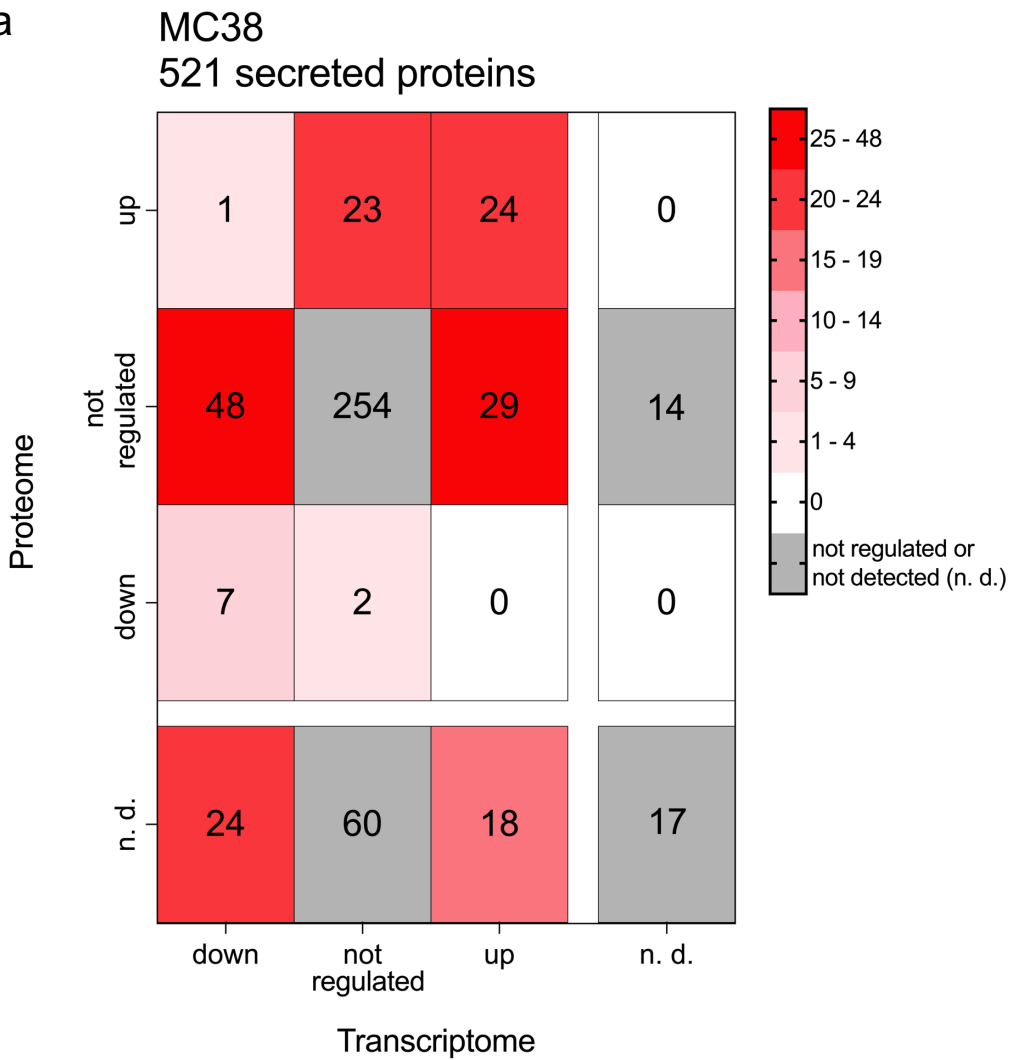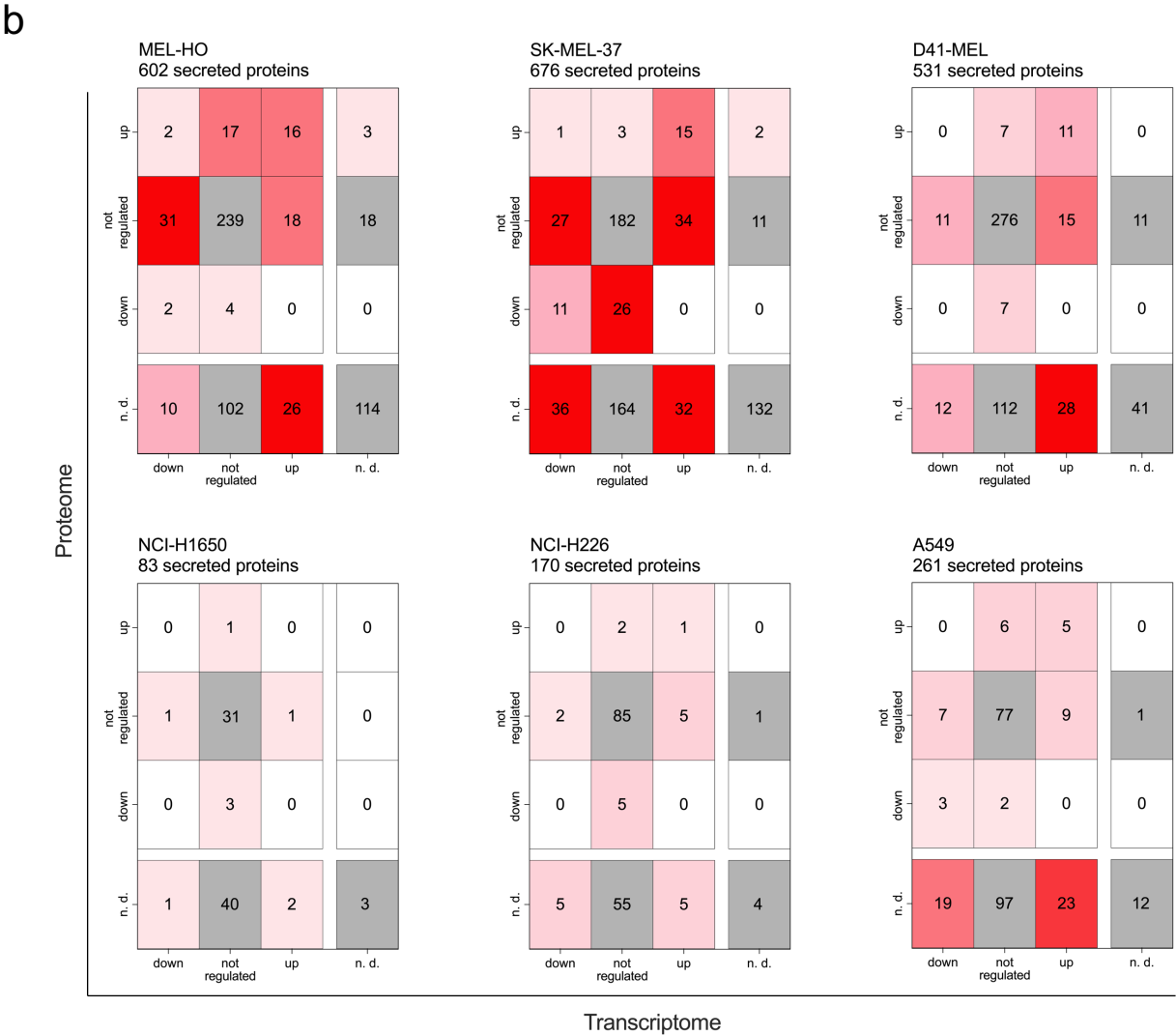

Supplement: Supplementary file 1 — Supplementary file1 (PDF 668 KB) [file 109_2023_2333_MOESM1_ESM.pdf]

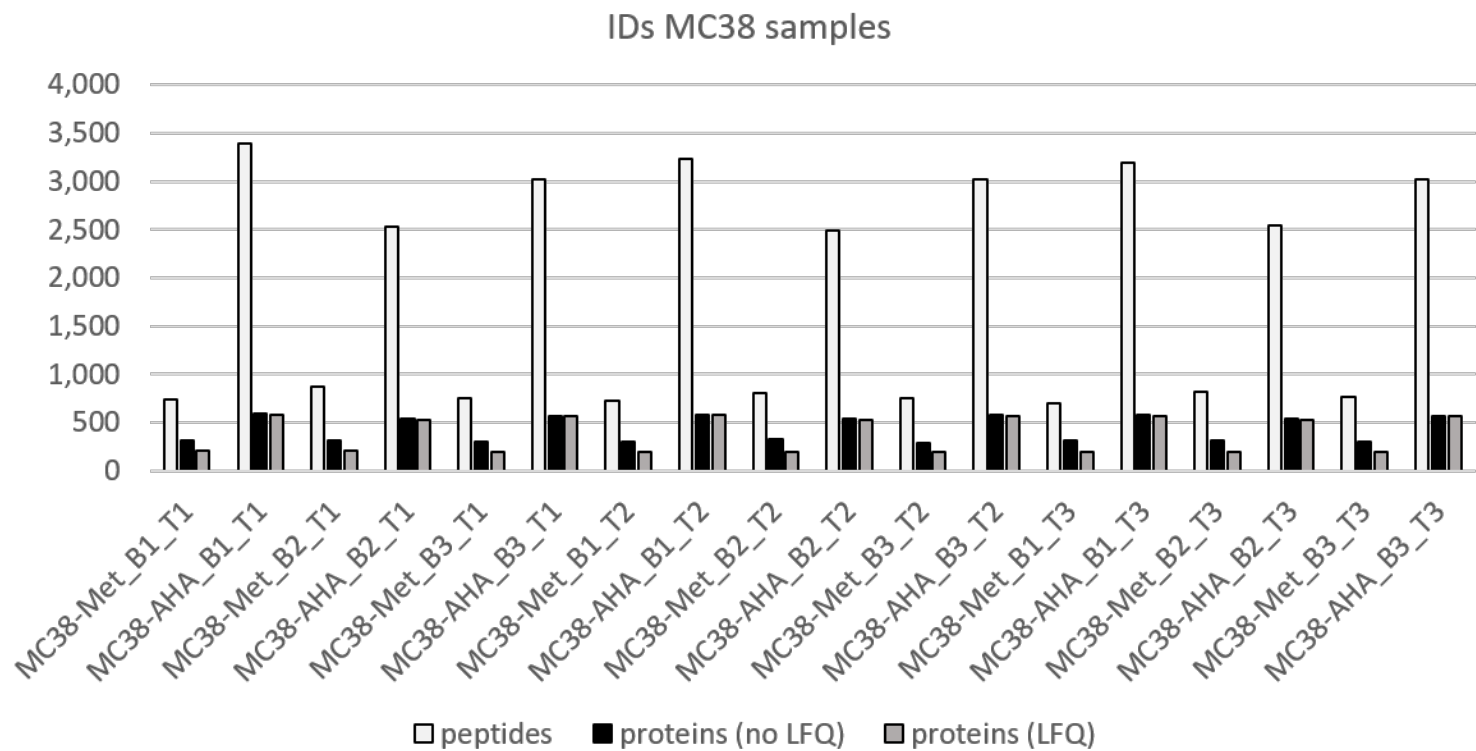

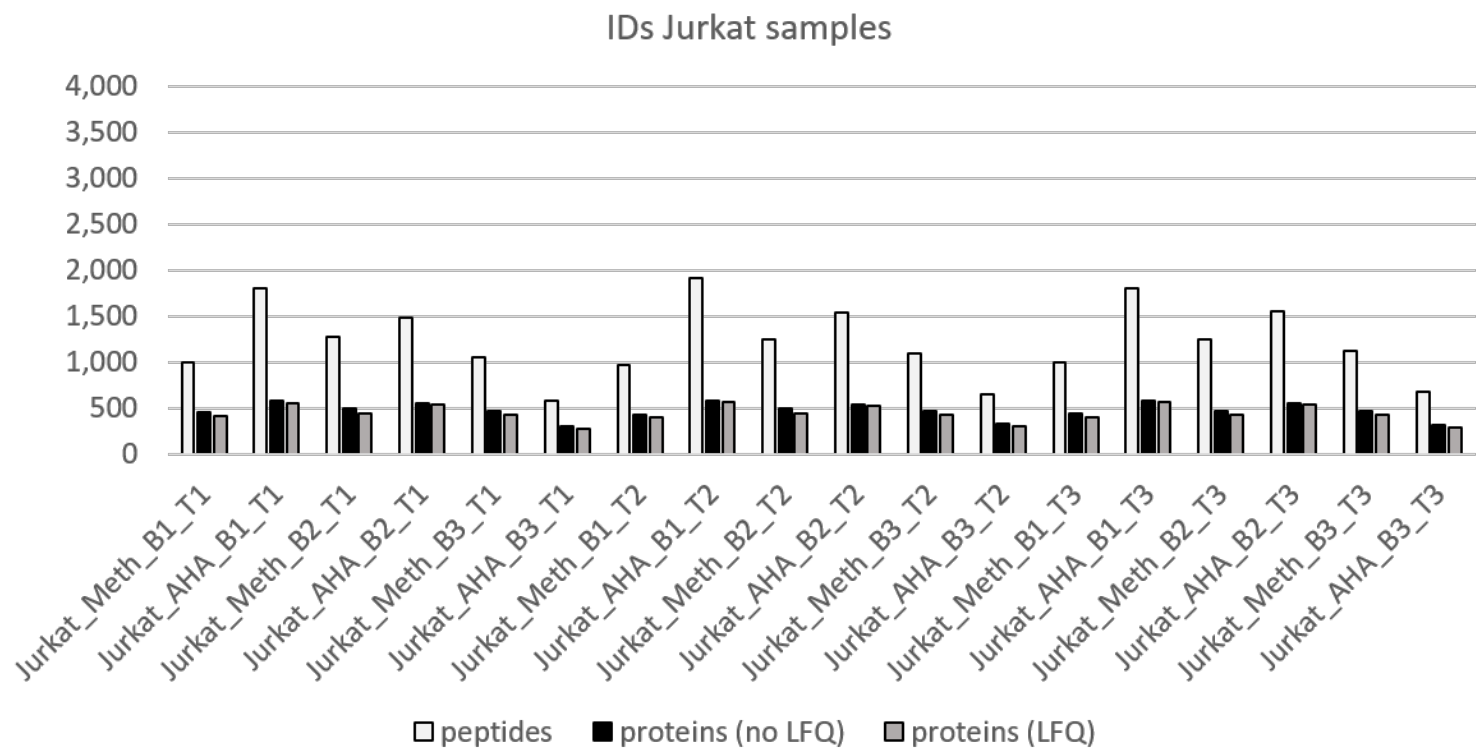

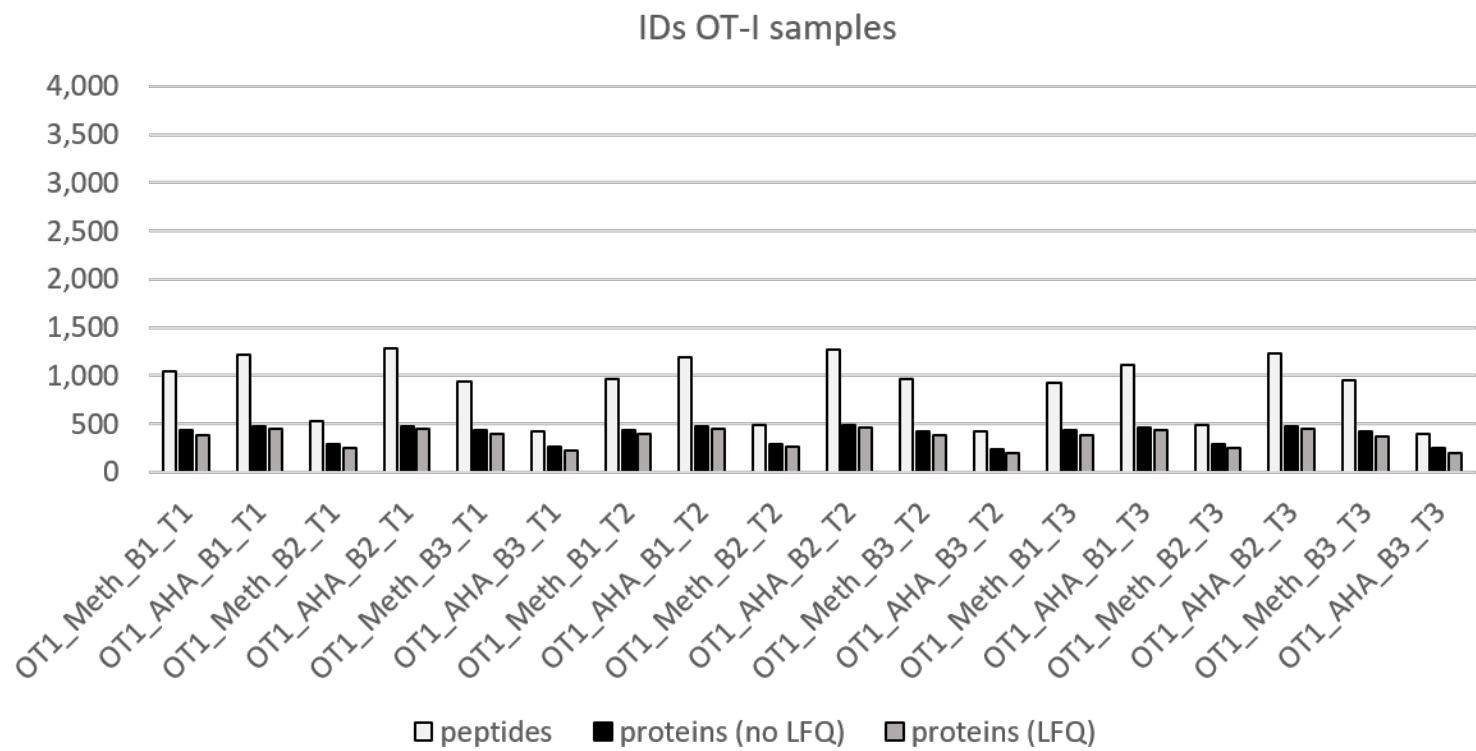

Supplement: Supplementary file 2 — Supplementary file2 (PDF 241 KB) [file 109_2023_2333_MOESM2_ESM.pdf]

# Supplementary Information SI3

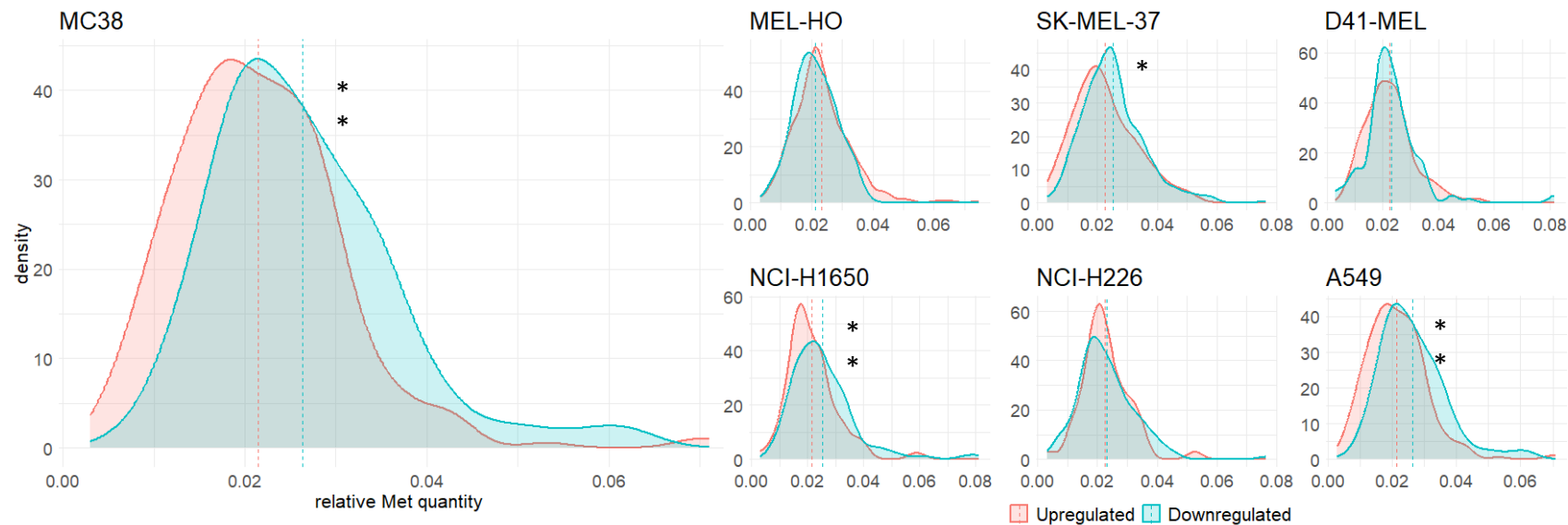

Supplement: Supplementary file 3 — Supplementary file3 (PDF 152 KB) [file 109_2023_2333_MOESM3_ESM.pdf]

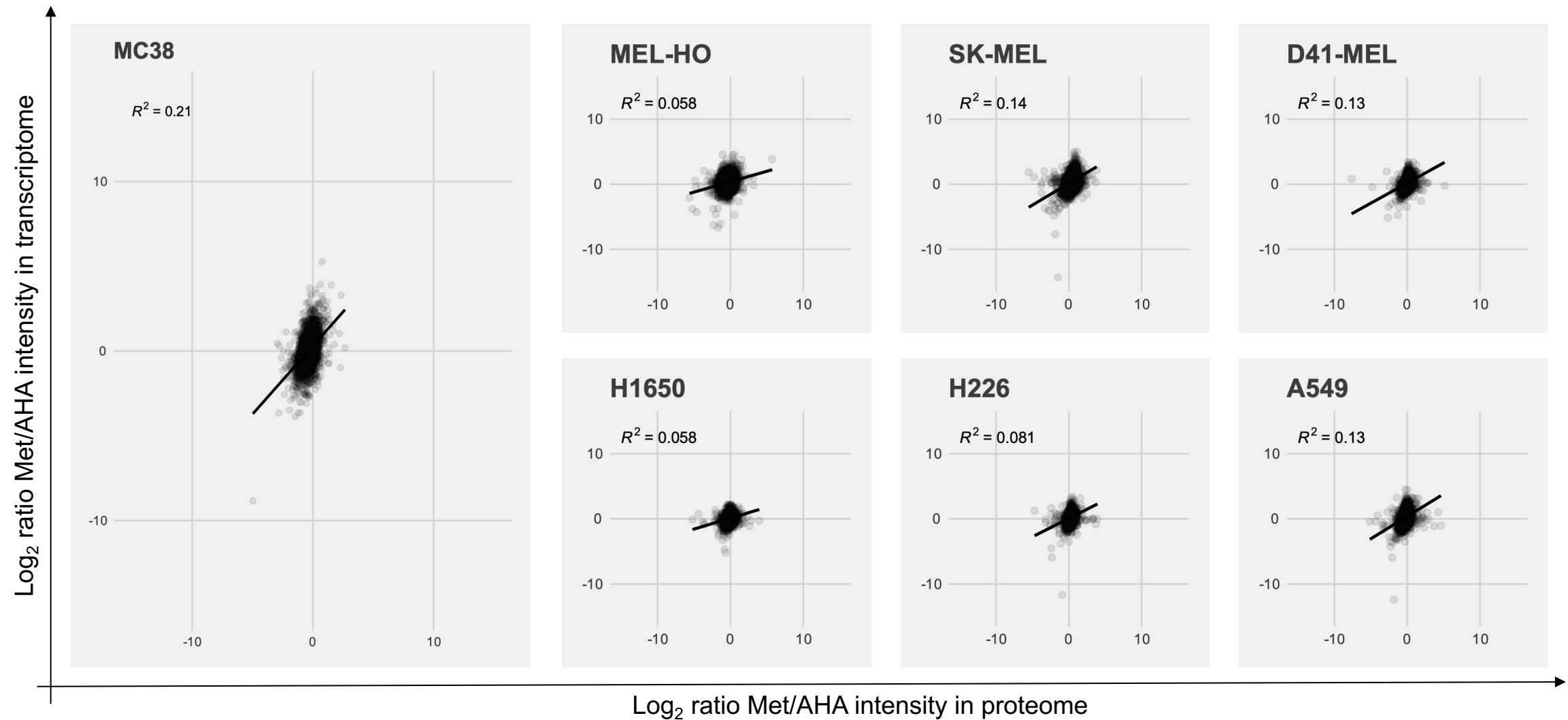

Supplement: Supplementary file 4 — Supplementary file4 (PDF 280 KB) [file 109_2023_2333_MOESM4_ESM.pdf]
